# Nomogram-based development and evaluation for predictions of 30-day and 1-year survival in patients with spontaneously ruptured hepatocellular carcinoma

**DOI:** 10.1186/s12885-022-10290-3

**Published:** 2022-11-15

**Authors:** Peng Wang, Shuping Yang, Chao Li, Xiangjun Han, Duo Hong, Haibo Shao

**Affiliations:** 1 Department of Interventional Radiology, the First Hospital of China Medical University, Shenyang, China; 2 Department of Pain Medicine, the First Hospital of China Medical University, Shenyang, China; 3 Department of Radiology, the Third Affiliated Hospital of Sun Yat-sen University, Guangzhou, China

**Keywords:** Hepatocellular carcinoma, Spontaneous rupture, Survival, Prediction model

## Abstract

**Background:**

Accurately predicting the prognosis of patients with spontaneously ruptured hepatocellular carcinoma (HCC) is crucial for effective clinical management. The aim of the present study was to establish and evaluate prediction models for 30-day and 1-year survival in patients with spontaneously ruptured HCC.

**Methods:**

A total of 118 patients with spontaneous rupture HCC were enrolled. Univariate and multivariate analyses were performed using logistic-regression model and Cox proportional-hazard model. The identified indicators were used to establish prediction models, the performance of which we compared with those of commonly used liver disease scoring models. The survival possibilities of different risk categories were calculated using the newly developed models.

**Results:**

Largest tumor size (LTS), serum albumin (ALB), total bilirubin (TBil), and serum creatinine were identified as independent predictors, which were used to establish a 30-day survival prediction model. LTS, BCLC staging, ALB, TBil, hepatectomy at rupture, and TACE during follow-up were identified as independent predictors of 1-year survival model. The 30-day survival model had sensitivity of 79.3%, specificity of 87.1%, and an AUC of 0.879, exhibiting better predictive performance than scores for Chronic Liver Failure Consortium Acute Decompensation score (CLIF-C ADs) and Model for End-stage Liver Disease (MELD). The 1-year survival model had sensitivity of 66.7%, specificity of 94.6%, and an AUC of 0.835, showing better predictive performance than Albumin–Bilirubin (ALBI), Child–Pugh, CLIF-C ADs, and MELD. After stratification, survival possibilities were 90.9 and 21.1% in low- and high-risk groups within 30 days, respectively, and 43.90, 4.35%, and 0 in low-, intermediate-, and high-risk groups at 1 year, respectively.

**Conclusions:**

The established models exhibited good performance in predicting both 30-day and 1-year survival in patients with spontaneously ruptured HCC.

## Introduction

Hepatocellular carcinoma (HCC) is one of the most common malignant tumors worldwide [1], with an incidence that ranks seventh and a mortality rate that ranks fourth [2] among all malignant tumors. Spontaneous rupture of HCC with intraperitoneal hemorrhage is a life-threatening complication. The incidence of spontaneous HCC rupture is 2.3–26% [3, 4], and mortality is as high as 25–75% [5] once rupture occurs. This condition is closely associated with poor prognosis, with a median survival less than time of 4 months [6].

Survival of patients with spontaneous rupture of HCC varies greatly. A number of researchers have made efforts to identify possible predictive factors, such as tumor stage, treatment methods, age, and Child–Pugh score, all of which are important in guiding clinicians to select appropriate management strategies for patients with spontaneous HCC rupture [7–9]. However, there is still no consensus on whether these different factors affect the short- and long-term survival rates of these patients [10]. In addition, the predictive values of models commonly used to evaluate patients with spontaneous rupture of HCC, including Child–Pugh score, Model for End-stage Liver Disease (MELD), Chronic Liver Failure Consortium Acute Decompensation score (CLIF-C ADs), and Albumin–Bilirubin (ALBI), are also unclear [6, 11].

We therefore conducted this retrospective study to identify risk factors and establish prediction models for short-term (30-day) and long-term (1-year) survival in patients with spontaneous rupture of HCC. Two nomograms were derived from the factors we identified to predict both survival rates for each patient. We determined and compared the performances of these two models using the commonly used liver disease scoring models Child–Pugh, MELD, CLIF-C ADs, and ALBI. Stratification of data by the two models enabled us to calculate and show the survival possibilities of patients in different risk categories.

## Materials and methods

### Patients

In this study, we enrolled 118 patients who suffered spontaneously ruptured HCC treated in our hospital during the period January 2010 to December 2020. Diagnosis of HCC followed Asia-Pacific clinical-practice guidelines on management of the disease [12]. HCC was diagnosed as chronic liver disease with at least two radiologically typical images showing distinctive HCC features based on dynamic contrast-enhanced computed tomography (CT), magnetic resonance imaging (MRI), or ultrasound (US); or with one radiological-imaging examination showing typical HCC features coupled with serum alpha-fetoprotein (AFP) > 400 ng/mL. Hemoperitoneum, perihepatic hematoma, extravasation of contrast agents, tumor protrusion from the hepatic surface, and localized discontinuity of the hepatic surface were all relevant imaging findings for diagnosis of ruptured HCC [13]. We recorded and retrospectively analyzed patients’ demographics, tumor characteristics, laboratory results, treatment methods, and follow-up data retrospectively. This study was conducted in accordance with the Declaration of Helsinki and approved by our hospital’s Ethics Committee.

### Treatments

All patients immediately received countershock treatments to ensure hemodynamic stability, including intravenous fluids and/or blood transfusions if necessary. After laboratory and imaging examinations were conducted, a multidisciplinary team evaluated patients’ conditions and administered appropriate treatments, which included hepatectomy, transarterial chemoembolization/transcatheter arterial embolization (TACE/TAE), and conservative treatment. Treatment of each patient was based on the patient’s tumor characteristics, liver function, and vital signs. A patient with stable hemodynamics, resectable tumor(s), and Child–Pugh class A liver function would be treated with hepatectomy. A patient with stable or unstable hemodynamics, unresectable tumor(s) or refusal of hepatectomy, and Child–Pugh class A/B liver function would be recommended to undergo TACE/TAE. For a patient with Child–Pugh class C (poor) liver function, conservative treatments would be suggested. All treatments were performed with the informed consent of the patient or his/her relatives.

### Hepatectomy

All surgeries were performed by experienced hepatobiliary surgeons. During surgery, the surgeon performed the Pringle maneuver with 10/5-min clamp/unclamp cycles. The infrahepatic inferior vena cava was closed during liver transection if severe bleeding from the major hepatic veins occurred. Kelly forceps and Erbe VIO bipolar forceps were used to perform hepatic parenchymal transection. Small-diameter vessels were electrocoagulated, while larger vessels were ligated. The primary purpose of surgery was hemostasis rather than R0 resection of tumors; R0 resection of all visible tumors would be expected only if safety were assured. If feasible, the surgeon aimed for a defined resection margin of > 1 cm. To avoid tumor cell seeding, the peritoneum was lavaged with a considerable amount of distilled water. And the hepatectomy is performed immediately rather than after TACE/TAE.

### TACE/TAE

After the artery supplying the tumor with blood and the bleeding location were identified by hepatic arteriography, the surgeon selectively inserted a microcatheter into the target vessel and delivered embolic materials. Patients with Child–Pugh class A liver function, stable hemodynamics, and no arterioportal or arteriovenous fistulas in the tumor(s) underwent TACE. Those with Child–Pugh class B liver function, hemodynamic instability, and arterioportal or arteriovenous fistulas in the tumor(s) received TAE. TACE was performed with emulsion of lipiodol and epirubicin, as well as embolic materials that included gelfoam and polyvinyl alcohol particles. After embolization, angiography was performed again to confirm the therapeutic effect.

### Conservative treatment

Patients who did not meet the criteria for surgical treatment and TACE/TAE and refused such treatments were treated conservatively, including with anti-shock therapy, correction of coagulopathy and liver function, and anti-infective therapy.

### Follow-up

We kept all patients under observation for the first month and then followed up on them every 3 months. Overall survival (OS) time was defined as the interval from the date of HCC rupture to the date of death or last follow-up. There was no patient experienced liver transplant and liver resection during follow-up period.

### Statistical analysis

Descriptive statistics are presented as means and standard deviations (SD) for normally distributed variables, or median and interquartile (IQR) ranges for non-normally distributed variables. Categorical variables are described as frequencies and percentages. We analyzed predictors using a logistic-regression model for the 30-day survival model and a Cox proportional-hazard model for the 1-year survival model. Predictors found to be significant were selected to establish new prediction models, which we compared with the commonly used models ALBI, Child–Pugh, CLIF-C ADs, and MELD. To facilitate practical use of the new models in predicting patient survival, we generated nomograms based on these models by fitting the models using the rms package in R software version 4.0.3 (http://www.R-project.org/). The models’ receiver operating characteristic (ROC) curves and areas under the curve (AUCs) were compared using the Delong test. We also plotted decision curves to assess the net benefit of model-assisted decisions. Additionally, we tested calibration, which determined whether predicted and observed probabilities were in agreement, by plotting predicted and observed survival and by using the Hosmer–Lemeshow goodness-of-fit test. We used X-tile software (version 3.6.1; Camp RL et al., 2004 [14]) to identify the optimal cutoff values of the 1-year survival prediction model and to separate patients into low-, intermediate- and high-risk groups. The cumulative survival rate was analyzed using the Kaplan–Meier method, and differences were compared using the log-rank test. *P* < 0.05 was considered statistically significant. Statistical analyses were performed using SPSS software version 21.0 (IBM Corp., Armonk, NY, USA) and R software.

## Results

### Demographic and clinical characteristics of patients

A total of 118 consecutive patients with ruptured HCC were enrolled in this study. Their demographic and clinical features at baseline are listed in Table 1. Mean age was 54 years, and 97 (82.20%) of patients were male. Ninety-five (80.51%) of patients were diagnosed with cirrhosis of the liver. According to contrast-enhanced CT/MRI results, 74 (62.2%) of ruptured tumors were located in the right lobe. Of all patients, 60 (50.4%), 43 (36.1%), and 16 (13.4%) were classified as Child–Pugh class A, B, or C, respectively. There were six patients receiving sorafenib in the follow-up period, of which one patient received TACE at HCC rupture and five patients underwent conservative treatment at rupture. Altogether, 94 (79.66%) of patients survived > 30 days, and 37 (31.36%) survived > 1 year. Median survival time was 174 (range, 45–479) days.

**Table 1:** Clinical characteristics of patients with spontaneously ruptured HCC

Index	Patients (***n*** = 118)	%
Age (years)	54 ± 11	
Gender		
Male	97	82.20
Female	21	17.80
Cirrhosis		
Yes	95	80.51
No	23	19.49
Viral hepatitis		
HBV	97	82.20
HCV	4	3.39
None	17	14.41
BCLC stage		
A	8	6.78
B	80	67.80
C	30	25.42
Ruptured-lesion location		
Left lobe	44	37.29
Right lobe	74	62.71
Treatment at rupture		
TAE/TACE	55	46.61
Hepatectomy	40	33.90
Conservative	23	19.49
Total number of tumors		
1	54	45.76
2–3	34	28.81
> 3	30	25.43
LTS (cm)	7.70 (5.45–10.93)	
Nodular (< 5 cm)	30	25.42
Massive (> 5 cm)	88	74.58
Vascular invasion		
No	88	74.58
Yes	30	25.42
Child–Pugh class		
A	59	50.00
B	43	36.44
C	16	13.56
MELD score	10.00 (8–13)	
≤ 14	95	80.5
14–18	15	12.7
> 18	8	6.8
Treatment during follow-up		
No	79	66.95
Yes	39	33.05
AFP (ng/ml)	427.60 (33.9–1210)	
≤ 400	56	47.46
> 400	62	52.54
Survival time > 30 days	94	79.66
Survival time > 1 year	37	31.36
Median survival time (days)	174 (45–479)	
Median follow-up time (days)	1292 (261–1405)	

*HBV* Hepatitis B virus, *HCV* Hepatitis C virus, *TAE* Transcatheter arterial embolization, *TACE* Transarterial chemoembolization, *LTS* Largest tumor size, *MELD* Model for End-stage Liver Disease

### Newly developed models for 30-day and 1-year survival

Of all enrolled factors, we identified four as independent predictors of 30-day survival: largest tumor size (LTS; cm), serum albumin (ALB; g/L), total bilirubin (TBil; umol/L), and serum creatinine (SCr; umol/L; Table 2). These independently associated risk factors were used as the basis of the 30-day survival model, described by the following formula:

$$\mathrm Y\;=\;0.182\;\times\;\mathrm{LTS}\;+\;-0.148\;\times\;\mathrm{ALB}\;+\;0.03\;\times\;\mathrm{TBil}\;+\;0.021\;\times\;\mathrm{SCr}\;-\;1.518$$

**Table 2:** Univariate and multivariate analyses of possible indicators related to 30-day survival

Variables	Univariate analysis			Multivariate analysis		
	OR	95% CI	***P***	OR	95% CI	***P***
Gender (male/female)	0.359	0.078–1.661	0.190			
Age (years)	1.026	0.985–1.069	0.220			
LTS (cm)	1.160	1.039–1.294	**0.008**	1.233	1.061–1.434	**0.006**
BCLC (A + B/C)	2.086	0.800–5.438	0.133			
Treatment at rupture						
Conservative (control)	–	–	–	–	–	–
TAE/TACE	0.338	0.115–0.997	**0.049**	0.560	0.104–3.017	0.500
Hepatectomy	0.229	0.065–0.805	**0.022**	0.811	0.141–4.655	0.814
Blood transfusion	0.500	0.154–1.627	0.250			
Viral hepatitis (none/HBV + HCV)	0.739	0.233–2.692	0.710			
Cirrhosis (no/yes)	1.892	0.512–6.988	0.339			
AFP (≤400/> 400)	1.085	0.442–2.667	0.858			
WBC (× 10^9^/L)	1.048	0.973–1.128	0.217			
HB, (g/L)	0.975	0.956–0.994	**0.009**	0.985	0.956–1.014	0.301
PT (s)	1.359	1.142–1.616	**0.001**	0.934	0.707–1.233	0.630
ALB (g/L)	0.866	0.804–0.933	**< 0.001**	0.873	0.786–0.969	**0.011**
TBil (umol/L)	1.031	1.012–1.051	**0.002**	1.033	1.005–1.062	**0.023**
Na^+^ (mmol/L)	0.912	0.825–1.007	0.069			
SCr (umol/L)	1.018	1.008–1.029	**0.001**	1.023	1.008–1.038	**0.002**

*OR* Odds ratio, *CI* Confidence interval, *LTS* Largest tumor size, *BCLC* Barcelona Clinic Liver Cancer score, *TAE* Transcatheter arterial embolization, *TACE* Transarterial chemoembolization, *HBV* Hepatitis B virus, *HCV* Hepatitis C virus, *AFP* Alpha-fetoprotein, *WBC* White blood cell count, *HB* Hemoglobin, *PT* Prothrombin time, *ALB* Albumin, *TBil* total bilirubin, *SCr* Serum creatinine

**Fig. 1:**
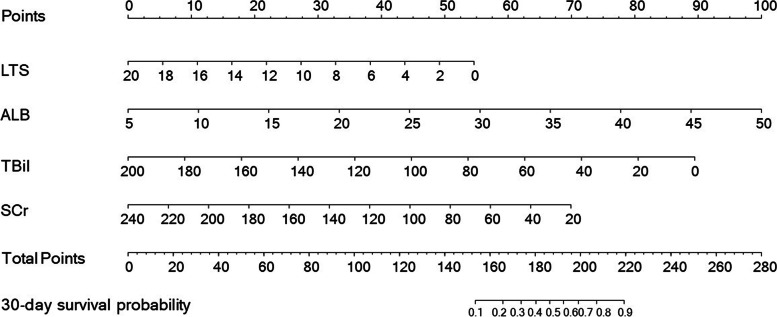
Nomogram of independent predictors of 30-day survival in patients with ruptured HCC. The nomogram was scaled by the proportional-regression coefficient of each predictor. LTS: largest tumor size (cm); ALB: albumin (g/L); SCr: serum creatinine (umol/L); TBil: total bilirubin (umol/L)

We established a nomogram that incorporated all significant prognostic factors (Fig. 1). Its scoring formula was as follows:

$${\mathrm{Points}}_{30-\mathrm{day}\;\mathrm{model}}\;=\;-2.73\;\times\;\mathrm{LTS}\;+\;54.57\;+\;2.22\;\times\;\mathrm{ALB}\;-\;11.11\;+\;-0.45\;\times\;\mathrm{TBil}\;+\;89.43\;+\;-0.32\;\times\;\mathrm{SCr}\;+\;76.23$$

Similarly, we identified six predictors as independent prognostic factors for OS: LTS (cm), Barcelona Clinic Liver Cancer (BCLC) staging, hepatectomy (HT) at rupture of HCC, TACE during follow-up (TDF), ALB, and TBil (Table 3). These independently associated risk factors were used to establish our 1-year survival model, described by the following formula:

$$\mathrm Y\;=\;0.08\;\times\;\mathrm{LTS}\;+\;0.623\;\times\;\mathrm{BCLC}\;+\;-1.079\;\times\;\mathrm{HT}\;+\;-0.932\;\times\;\mathrm{TDF}\;+\;0.015\;\times\;\mathrm{TBil}\;+-0.047\;\times\;\mathrm{ALB}$$

**Table 3:** Univariate and multivariate analyses of possible indicators related to overall survival

Variables	Median survival time (days)	Univariate analysis			Multivariate analysis		
		HR	95% CI	***P***	HR	95% CI	***P***
Gender (male/female)	170/255	0.668	0.362–1.234	0.198	1.289	0.628–2.644	0.489
Age (years)		1.005	0.985–1.026	0.603	0.992	0.970–1.016	0.517
LTS (cm)		1.074	1.025–1.126	**0.003**	1.086	1.021–1.154	**0.008**
BCLC (A + B/C)	239/59	2.798	1.748–4.481	**< 0.001**	1.095	1.076–3.372	**0.027**
Treatment at rupture							
Conservative (control)	48	–	–	–	–	–	–
TAE/TACE	159	0.565	0.334–0.956	**0.033**	0.660	0.354–1.231	0.192
Hepatectomy	466	0.202	0.105–0.388	**< 0.001**	0.180	0.076–0.427	**< 0.001**
Blood transfusion (no/yes)	160/381	0.495	0.287–0.854	**0.012**	1.053	0.540–2.052	0.880
Viral hepatitis (none/HBV + HCV)	184/172	0.977	0.530–1.803	0.941			
Cirrhosis (no/yes)	207/168	1.394	0.783–2.482	0.259			
AFP (≤400/> 400)		1.549	0.994–2.412	0.053			
WBC (×10^9^/L)		0.992	0.953–1.032	0.686			
HB (g/L)		0.998	0.989–1.007	0.642			
PT (s)		1.137	1.056–1.224	**0.001**	0.994	0.845–1.055	0.312
ALB (g/L)		0.959	0.932–0.988	**0.005**	0.944	0.905–0.984	**0.007**
TBil (umol/L)		1.015	1.008–1.021	**< 0.001**	1.021	1.010–1.032	**< 0.001**
Na^+^ (mmol/L)		0.945	0.892–1.000	**0.049**	1.040	0.971–1.113	0.266
SCr (umol/L)		1.003	0.998–1.009	0.213			
TACE during follow-up (no/yes)	92/347	0.420	0.255–0.692	**0.001**	0.414	0.233–0.737	**0.003**

*OR* Odds ratio, *CI* Confidence interval, *LTS* Largest tumor size, *BCLC* Barcelona Clinic Liver Cancer score, *TAE* Transcatheter arterial embolization, *TACE* Transarterial chemoembolization, *HBV* Hepatitis B virus, *HCV* Hepatitis C virus, *AFP* Alpha-fetoprotein, *WBC* White blood cell count, *HB* Hemoglobin, *PT* Prothrombin time, *ALB* Albumin, *TBil* total bilirubin, *SCr* Serum creatinine

**Fig. 2:**
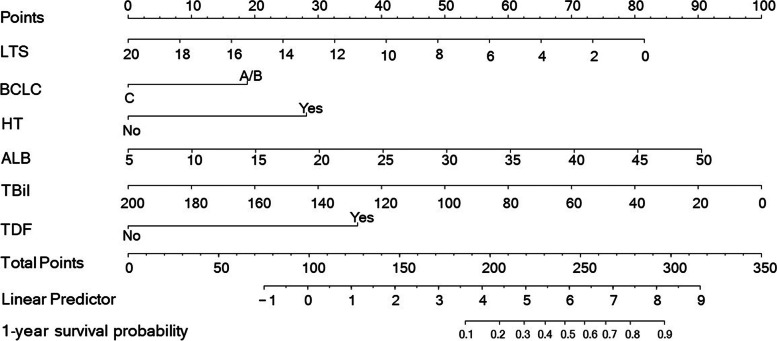
Nomogram of independent predictors of 1-year survival in patients with ruptured HCC. The model was presented with a nomogram scaled by the proportional regression coefficient of each predictor. LTS: largest tumor size (cm); BCLC: Barcelona Clinic Liver Cancer score; HT: hepatectomy; ALB: albumin (g/L); TBil: total bilirubin (umol/L); TDF: TACE during follow-up

We established a nomogram that incorporated all significant prognostic factors (Fig. 2). Its scoring formula was as follows:

$${\mathrm{Points}}_{1-\mathrm{year}\;\mathrm{model}}\;=\;-4.08\;\times\;\mathrm{LTS}\;+\;81.56\;+\;18.81\;\times\;\mathrm{BCLC}\;(\mathrm{BCLC}\;\mathrm A/\mathrm B\;=\;1,\;\mathrm C\;=\;0)\;+\;36.24\;\times\;\mathrm{TDF}\;(\mathrm{Yes}\;=\;1,\;\mathrm{No}\;=\;0)\;+\;28.11\;\times\;\mathrm{HT}\;(\mathrm{Yes}\;=\;1,\;\mathrm{No}\;=\;0)\;+\;2.01\;\times\;\mathrm{ALB}\;-\;10.06\;+\;-0.5\;\times\;\mathrm{TBil}\;+\;100.$$

### Performance of the prediction models

**Fig. 3:**
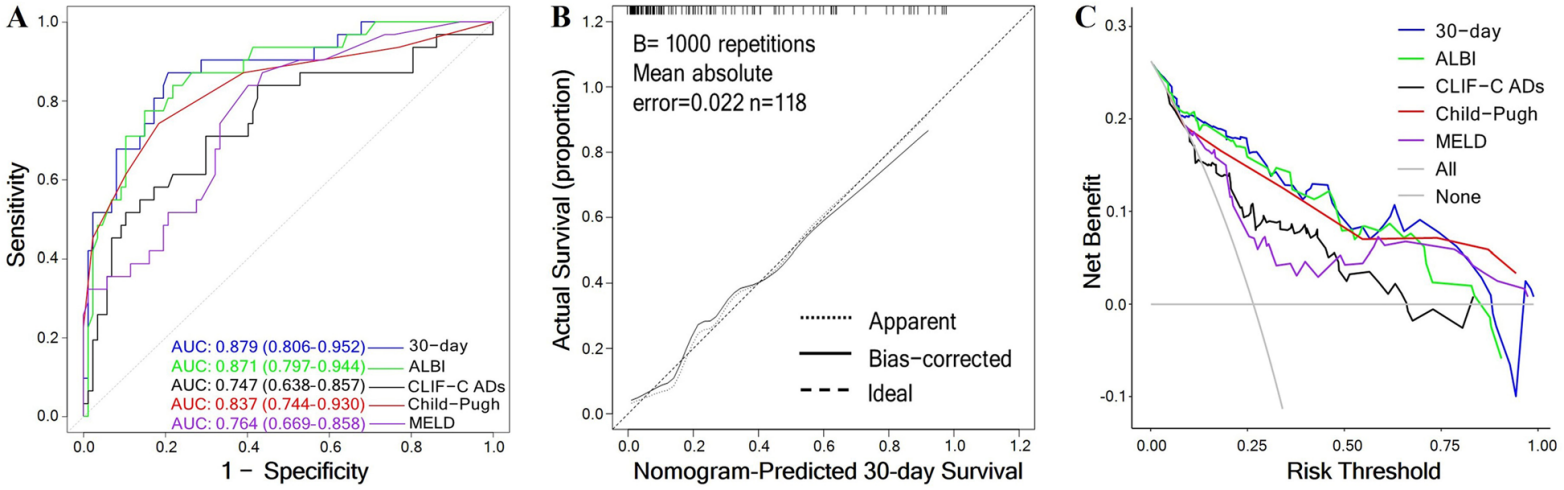
Performance of the 30-day survival model in patients with ruptured HCC. **A** Receiver operating characteristic (ROC) curves of different models. **B** Calibration plot of 30-day survival model in patients with ruptured HCC. **C** Decision curve analyses of different models

The 30-day survival model had sensitivity of 79.3%, specificity of 87.1%, and an AUC of 0.879 (95% confidence interval [CI], 0.806–0.952), performing similarly to ALBI (AUC, 0.871; 95% CI, 0.797–0.944; *P* = 0.574) and Child–Pugh (AUC, 0.837; 95% CI, 0.744–0.930; *P* = 0.2) but better than CLIF-C ADs (AUC, 0.747; 95% CI, 0.638–0.857; *P* = 0.01) and MELD (AUC, 0.764; 95% CI, 0.669–0.858; *P* = 0.007; Fig. 3A, Table 4). The model’s calibration curve demonstrated good agreement between predicted and observed 30-day survival rates (Hosmer–Lemeshow, χ^2^ = 3.91, *P* = 0.86; Fig. 3B), which indicating the model based on nomograph is reliable and accurate. Decision curve analysis showed that the net benefit of the 30-day model was superior to those of the CLIF-C ADs and MELD models (Fig. 3C).

**Table 4:** Performance of the 30-day survival model and other commonly used models

Models	AUC	95% CI	***P***	Sensitivity	Specificity
30-day model	0.879	0.806–0.952	–	79.3	87.1
ALBI score	0.871	0.797–0.944	0.574	77.4	85.1
CLIF-C ADs	0.747	0.638–0.857	**0.010**	83.9	57.5
Child–Pugh score	0.837	0.744–0.930	0.200	74.2	81.6
MELD	0.764	0.669–0.858	**0.007**	83.9	59.8

*CI* Confidence interval, *ALBI* Albumin–Bilirubin, *CLIF-C ADs* Chronic Liver Failure Consortium Acute Decompensation score, *MELD* Model for End-stage Liver Disease

**Fig. 4:**
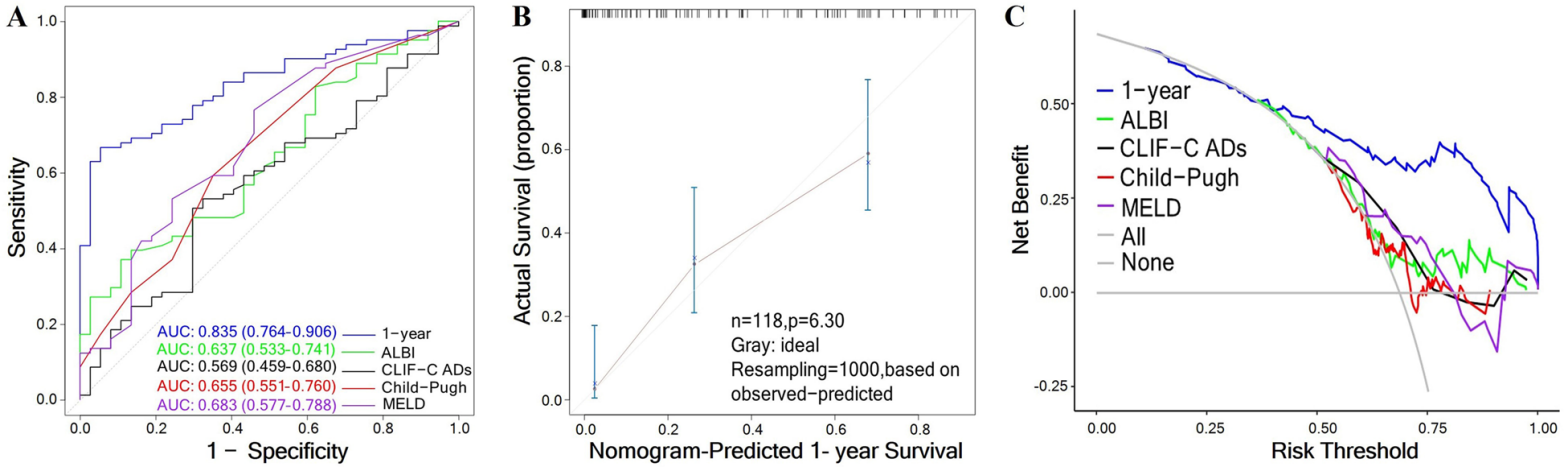
Performance of the 1-year survival model in patients with ruptured HCC. **A** Receiver operating characteristic (ROC) curves of different models. **B** Calibration plot of the 1-year model in patients with ruptured HCC. **C** Decision curve analyses of different models

The 1-year survival model had sensitivity of 66.7%, specificity of 94.6%, and an AUC of 0.835 (95% CI, 0.764–0.906), which showed better predictive performance than ALBI (AUC, 0.637; 95% CI, 0.533–0.741; *P* < 0.001), Child–Pugh (AUC, 0.655; 95% CI, 0.551–0.760; *P* < 0.001), CLIF-C ADs (AUC, 0.569; 95% CI, 0.459–0.680; *P* < 0.001), and MELD (AUC, 0.683; 95% CI, 0.577–0.788; *P* = 0.004; Fig. 4A, Table 5). The 1-year model showed satisfactory calibration (Fig. 4B), which indicating the model based on nomograph is reliable and accurate. Decision curve analysis showed that the net benefit of the 1-year survival model was superior to that of each of the abovementioned models (Fig. 4C).

**Table 5:** Performance of the 1-year survival model and other commonly used models

Models	AUC	95% CI	***P***	Sensitivity	Specificity
1-year model	0.835	0.764–0.906	–	66.7	94.6
ALBI score	0.637	0.533–0.741	**< 0.001**	37.0	89.2
Child–Pugh score	0.655	0.551–0.760	**< 0.001**	59.3	64.9
CLIF-C ADs	0.569	0.459–0.680	**< 0.001**	50.6	70.3
MELD	0.683	0.577–0.788	**0.004**	76.5	54.1

*CI* Confidence interval, *ALBI* Albumin–Bilirubin, *CLIF-C ADs* Chronic Liver Failure Consortium Acute Decompensation score, *MELD* Model for End-stage Liver Disease

### Risk stratification

**Fig. 5:**
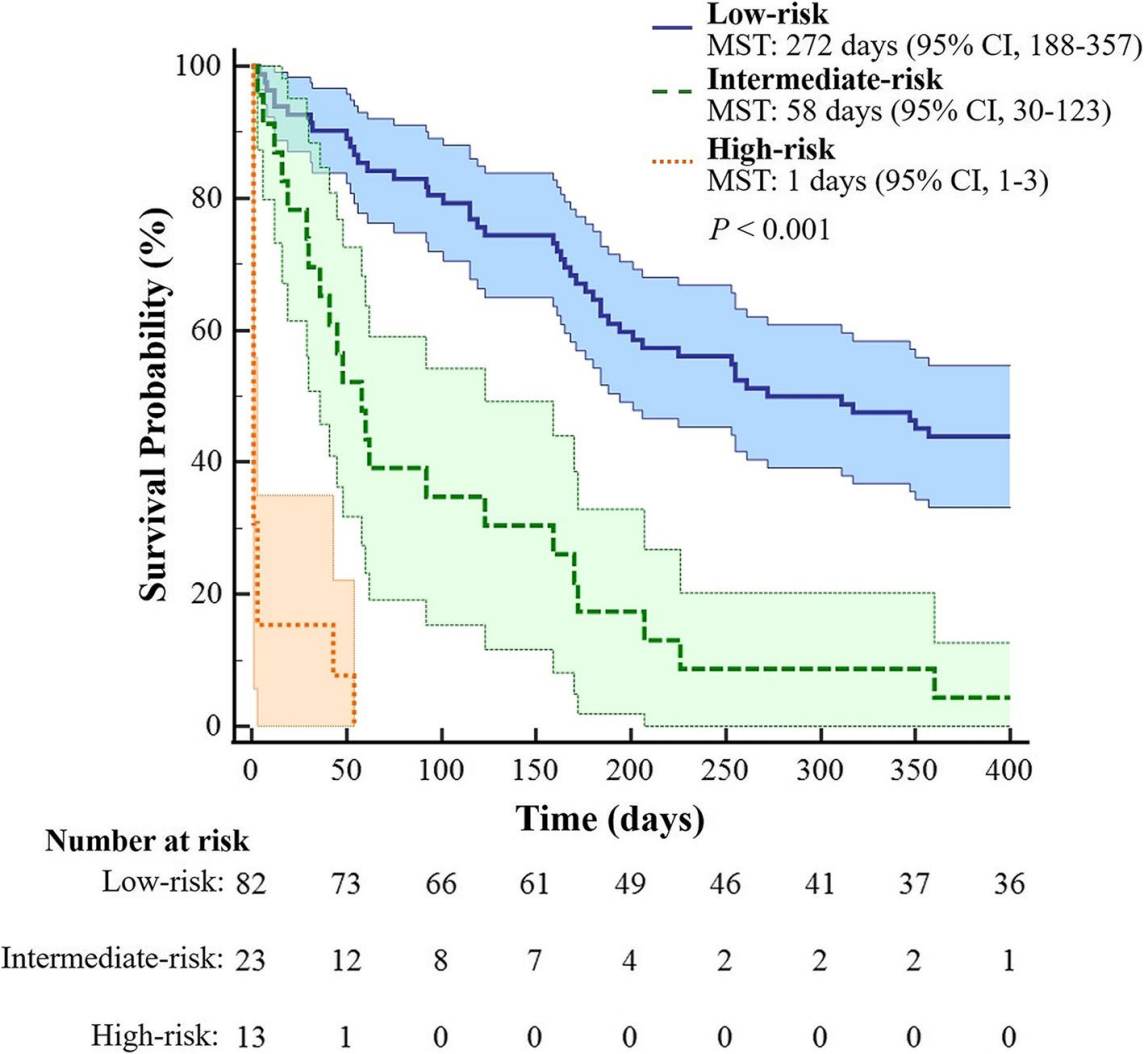
Risk stratification according to nomogram scores of 1-year model. For the 1-year model risk classification, low-, intermediate-, and high-risk score values were > 204.8, 159.9–204.8, and < 159.9, respectively. Median survival times were 272 days (95% CI, 188–357), 58 days (95% CI, 30–123), and 1 day (95% CI, 1–3), and probabilities of 1-year survival were 43.90, 4.35, and 0%, in the low-, intermediate-, and high-risk groups, respectively. MST: median survival time

Patients were stratified into different risk categories based on the newly developed models. For the 30-day survival model, we obtained a cutoff score value of 188.5 using the maximum Youden index. Survival possibility within 30 days after rupture of HCC was 90.9% (score value < 188.5, *n* = 99) in the low-risk group and 21.1% in the high-risk group (score value ≤188.5, *n* = 19; χ^2^ = 43.80, *P* < 0.001). For the 1-year survival model, we used X-tile plots to generate the two optimal cutoff scores of 159.9 and 204.8, which separated patients into the three groups with highly different probabilities of survival. Estimated median-survival times were 272 days (95% CI, 188–357), 58 days (95% CI, 30–123), and 1 day (95% CI, 1–3), and 1-year cumulative survival rates were 43.90, 4.35%, and 0, in the low-risk (score value > 204.8, *n* = 82), intermediate-risk (score value 159.9–204.8, *n* = 23) and high-risk (score value < 159.9, *n* = 13) groups, respectively (*P* < 0.001; Fig. 5).

## Discussion

As a life-threatening complication of HCC, spontaneous tumor rupture always causes patient prognosis to deteriorate and poses a high rate of short-term death [15]. So far, the search for predictors of survival in these patients, in which numerous efforts have been made, has failed to reach consensus. However, recognition of such prognostic factors is critical for physicians to manage patients with ruptured HCC. In our cohort study, the median-survival time of HCC rupture patients was 174 days, in which the 30-day survival rate (short-term survival) was 79.66% and the 1-year survival rate (long-term survival) was 31.36%. We analyzed the factors related to short- and long-term survival in these patients using a logistic-regression model and a Cox regression model. The results showed that LTS, BCLC stages, hepatectomy at rupture, follow-up TACE after rupture (TAR), TBil, and ALB were predictors of 1-year survival in ruptured HCC. Similarly, LTS, ALB, TBil, and SCr were valuable predictors of 30-day survival. Some of our findings have been analyzed in previous studies. Han et al. considered TBil and BCLC stages as independent predictors of poor survival in patients with HCC rupture [6]. Zhang et al. showed that hepatectomy was a good choice for improving survival and preserving liver function in these patients [11]. In addition, as in our study, some previous studies have demonstrated that TAR is inversely associated with poor survival. Both TACE and TAE have high success rates of hemostasis, but recent studies show that TACE cannot improve survival due to its adverse effect of liver function injury [16–18]. Tumor size and number and ALB level have been considered predictors of ruptured HCC in previous studies [13, 19]. The results of the current study also showed that ALB level was the only protective biochemical index. However, whether a large amount of albumin supplementation can improve patient prognosis was unclear. Furthermore, we found that SCr was closely associated with poor 30-day survival, which might be attributable to severe impairment of renal function caused by massive bleeding.

Previous studies have shown very poor prognosis in patients with HCC rupture, with a 30-day mortality rate of 30–70% [20, 21] and 1-year mortality rate of nearly 40% [22]. Fortunately, recent reports have indicated a significant decrease in the mortality rate [13]. In this study, we observed a 30-day mortality rate of 20.34% and a 1-year mortality rate of 68.64%. Some studies have demonstrated that liver function indices such as TBil, ALB, and prothrombin time (PT) are significant predictors [6, 23]. In this study, we investigated the predictive value of four existing liver function evaluation models for the prognoses of HCC rupture patients that have rarely been examined in previous studies: ALBI, Child–Pugh score, CLIF-C ADs, and MELD. Child–Pugh is the most widely used scoring model in clinical practice [24]; unlike the other three, it contains two subjective indices of ascites and encephalopathy, both of which can be corrected by therapy. ALBI, first proposed by Johnson et al. [25] in 2015, considers albumin and bilirubin levels. Recent studies show that ALBI has better predictive effectiveness in evaluating the prognosis of patients with HCC than Child–Pugh [26, 27]. MELD considers the continuous functions of bilirubin, international normalized ratio (INR), and SCr to predict survival rates in patients with end-stage liver disease [28]. The MELD score was developed to evaluate patients undergoing transjugular intrahepatic portosystemic shunt (TIPS) [29]. Jalan et al.’s prospective study showed that CLIF-ADs, a model based on age, white blood cell (WBC) count, SCr, INR, and sodium, accurately predicted the risk of mortality in patients with acute decompensation of liver disease [30]. In the present study, the results showed no significant differences in 30-day or 1-year survival predictive performance among the abovementioned four models (*P* > 0.05). However, we found that Child–Pugh might not suitable for predicting prognosis in patients with HCC rupture, because distinguishing whether ascites is caused by liver dysfunction or tumor rupture hemorrhage is difficult. In terms of risk stratification, by dividing patients into low- (score value > 204.8), intermediate- (159.9–204.8), and high-risk (< 159.9) groups, we were able to show a very marked gradient of 1-year survival probability. Going from low to intermediate risk, the 1-year survival probability decreased by a factor of 10; going from intermediate to high risk, this probability decreased to 0. In addition, according to the calculation formula of the 1-year model score, we could find that patients receiving hepatectomy at HCC rupture can increase the score and be divided into low-risk groups. Hence, once patients are classified into low-risk groups, hepatectomy should be selected at first. However, the patients in the high-risk group have a very high mortality rate in the short term due to unstable vital signs and inability to receive effective treatment in time. Therefore, our data showed that the 1-year prediction model was useful for identifying patients at all three levels of risk; thus, HCC rupture, as a serious complication with high mortality, needs intense attention and active treatment.

Based on independently associated risk factors, we then established 30-day and 1-year models to predict 30-day and 1-year survival rates, respectively, in patients with HCC. C-index and decision curve analysis results demonstrated that the 1-year predictive model showed more precise predictive ability than ALBI, Child–Pugh, MELD, and CLIF-C ADs. From our perspective, the 1-year model contained more information, including treatment, tumor size and stages, and biochemical indices, than the abovementioned four models. Similarly, the 30-day model also showed more-powerful predictive efficacy than either MELD or CLIF-C ADs.

This study has several limitations. First, our study design was retrospective, raising the possibility of selection bias. Second, the sample size was not large enough. Third, validation of the models we presented was not conducted in an external cohort. A large-scale multicenter investigation might be required for such validation.

## Conclusions

We used independently associated risk factors to establish 30-day and 1-year models that demonstrated good performance in predicting clinical outcomes in patients with HCC rupture. These novel prediction models may provide clinicians with useful guidance for effective clinical management.

## Data Availability

The datasets generated and/or analysed during the current study are not publicly available due do not disclose publicly personal specific information has been promised in informed consent but are available from the corresponding author on reasonable request.

## Abbreviations

ALB: Albumin
ALT: Alanine transaminase
APTT: Activated partial thromboplastin time
AUC: Area under the curve
BCLC: Barcelona Clinic Liver Cancer
Cr: Creatinine
CT: Computed tomography
ECOG: Eastern cooperative oncology group
MRI: Magnetic resonance imaging
HB: Hemoglobin
HBV: Hepatitis B virus
HCC: Hepatocellular carcinoma
HCV: Hepatitis C virus
HR: Hazard ratio
HT: Hepatectomy
INR: International normalized ratio
LTS: Largest tumor size
MELD: Model for end-stage liver disease
OS: Overall survival
PLT: Platelet count
PT: Prothrombin time
PTA: Prothrombin activity
RBC: Red blood cell count
ROC: Receiver operating characteristic
TACE: Transarterial chemoembolization
TAE: Transcatheter arterial embolization
TBil: Total bilirubin
WBC: White blood cell count
